# Regulatory Architecture of the LβT2 Gonadotrope Cell Underlying the Response to Gonadotropin-Releasing Hormone

**DOI:** 10.3389/fendo.2018.00034

**Published:** 2018-02-14

**Authors:** Frederique Ruf-Zamojski, Miguel Fribourg, Yongchao Ge, Venugopalan Nair, Hanna Pincas, Elena Zaslavsky, German Nudelman, Stephanie J. Tuminello, Hideo Watanabe, Judith L. Turgeon, Stuart C. Sealfon

**Affiliations:** ^1^Department of Neurology, Center for Advanced Research on Diagnostic Assays, Icahn School of Medicine at Mount Sinai, New York, United States; ^2^Department of Medicine, Division of Pulmonary, Critical Care and Sleep Medicine, Icahn School of Medicine at Mount Sinai, New York, United States; ^3^University of California, Davis, Davis, CA, United States; ^4^Departments of Neuroscience and Pharmacological Sciences, Friedman Brain Institute, Icahn School of Medicine at Mount Sinai, New York, United States

**Keywords:** LβT2, gonadotrope, gonadotropin-releasing hormone, chromatin accessibility mapping, transcription profiling, single-cell transcriptomics

## Abstract

The LβT2 mouse pituitary cell line has many characteristics of a mature gonadotrope and is a widely used model system for studying the developmental processes and the response to gonadotropin-releasing hormone (GnRH). The global epigenetic landscape, which contributes to cell-specific gene regulatory mechanisms, and the single-cell transcriptome response variation of LβT2 cells have not been previously investigated. Here, we integrate the transcriptome and genome-wide chromatin accessibility state of LβT2 cells during GnRH stimulation. In addition, we examine cell-to-cell variability in the transcriptional response to GnRH using Gel bead-in-Emulsion Drop-seq technology. Analysis of a bulk RNA-seq data set obtained 45 min after exposure to either GnRH or vehicle identified 112 transcripts that were regulated >4-fold by GnRH (FDR < 0.05). The top regulated transcripts constitute, as determined by Bayesian massive public data integration analysis, a human pituitary-relevant coordinated gene program. Chromatin accessibility [assay for transposase-accessible chromatin with high-throughput sequencing (ATAC-seq)] data sets generated from GnRH-treated LβT2 cells identified more than 58,000 open chromatin regions, some containing notches consistent with bound transcription factor footprints. The study of the most prominent open regions showed that 75% were in transcriptionally active promoters or introns, supporting their involvement in active transcription. *Lhb, Cga*, and *Egr1* showed significantly open chromatin over their promoters. While *Fshb* was closed over its promoter, several discrete significantly open regions were found at −40 to −90 kb, which may represent novel upstream enhancers. Chromatin accessibility determined by ATAC-seq was associated with high levels of gene expression determined by RNA-seq. We obtained high-quality single-cell Gel bead-in-Emulsion Drop-seq transcriptome data, with an average of >4,000 expressed genes/cell, from 1,992 vehicle- and 1,889 GnRH-treated cells. While the individual cell expression patterns showed high cell-to-cell variation, representing both biological and measurement variation, the average expression patterns correlated well with bulk RNA-seq data. Computational assignment of each cell to its precise cell cycle phase showed that the response to GnRH was unaffected by cell cycle. To our knowledge, this study represents the first genome-wide epigenetic and single-cell transcriptomic characterization of this important gonadotrope model. The data have been deposited publicly and should provide a resource for hypothesis generation and further study.

## Introduction

Gonadotropin-releasing hormone (GnRH) plays a key role in the control of reproduction in mammals. Secreted by the hypothalamus in a pulsatile fashion, GnRH acts *via* its receptor (GnRHR) to trigger the synthesis and release of the luteinizing hormone (LH) and follicle-stimulating hormone (FSH) by the pituitary gonadotropes. In turn, the gonadotropins regulate gametogenesis and steroidogenesis in the gonads. The gonadotropins are composed of a common glycoprotein hormone α subunit (CGA) and a specific β subunit (LHβ or FSHβ). The frequency of GnRH pulse release varies at different stages of reproductive life, e.g., during puberty and the female menstrual cycle. GnRH pulse frequency differentially regulates gonadotropin subunit gene expression and gonadotropin secretion (1). While *Lhb* gene expression is preferentially induced by high-frequency GnRH pulses, low-frequency pulses favor *Fshb* expression (2, 3).

The immortalized LβT2 gonadotrope cells have been used extensively as an *in vitro* model for the study of gonadotropin gene regulation and GnRH signaling. The cell line was developed through targeted tumorigenesis in mice carrying the rat LHβ regulatory region linked to the SV40 T-antigen oncogene (4–6). LβT2 cells have some functional characteristics of mature gonadotropes, as they express *Cga, Gnrhr*, and *Lhb*. The cell line responds to pulsatile GnRH stimulation by upregulating *Lhb* and *Gnrhr* and secreting LH. In the presence of steroid hormones, LβT2 cells further increase the LH secretory response to GnRH pulses as well as the levels of *Lhb* and *Gnrhr* mRNAs (6). In addition, LβT2 cells induce *Fshb* under either activin A (7, 8) or GnRH pulse stimulation (3), with the level of *Fshb* being influenced by both pulse frequency and average concentration of GnRH (9). While LβT2 cells exhibit an increase in intracellular calcium and exocytosis in response to GnRH stimulation (5, 6), they differ from mature anterior pituitary cells in that they lack a characteristic large-amplitude calcium oscillatory response to GnRH (10). In addition, continuous GnRH stimulation does not induce *Gnrhr* gene expression, which is in contrast with rat pituitary cells (11).

Previous studies in LβT2 cells showed that GnRH activates a complex cell signaling network that rapidly induces the expression of early genes such as *Egr1, c-Fos*, and *c-Jun* (12–14), whose products consecutively activate the transcription of gonadotropin subunit genes. Over the past two decades, a number of studies in the LβT2 cell line have implicated various pituitary factors in gonadotropin subunit gene regulation. These factors include secreted peptides such as bone morphogenetic proteins, pituitary adenylate cyclase-activating polypeptide, growth differentiation factor 9, VGF nerve growth factor inducible (15–19) [for review, see Ref. (20)], as well as transcription factors (TFs) such as AP1 (Fos/Jun heterodimer), SF1, and Egr1 (14, 21–23). Nevertheless, the molecular mechanisms underlying the gonadotrope response to GnRH and the decoding of the GnRH pulse signal are not fully understood.

Recent advances in high-throughput sequencing technologies have enabled researchers to solve key questions about gene regulation both at the chromatin and at the transcriptome levels. Hence, mapping of “open” chromatin regions using the assay for transposase-accessible chromatin with high-throughput sequencing (ATAC-seq) allows the detection of putative DNA regulatory regions that are likely bound by TFs (24, 25). Correlating the transcriptome (measured by RNA-seq) with a map of open chromatin may identify transcriptional regulatory elements that are involved in the GnRH response. Furthermore, as distinct cells within a cell population display significant variations in RNA expression, analysis of cell-to-cell variability of gene expression can deepen our understanding of cell population complexity and transcriptome dynamics by isolating transcriptomic heterogeneity (e.g., cell cycle status) that is concealed in cell population studies [for reference, see Ref. (26)] and providing insight into the cellular variation in gene expression levels and induction. Gel bead-in-emulsion (GEM) Drop-seq is a droplet-based single-cell (SC) RNA-seq method that can profile thousands of individual cells per sample with high sensitivity (27–29).

**Figure 1:**
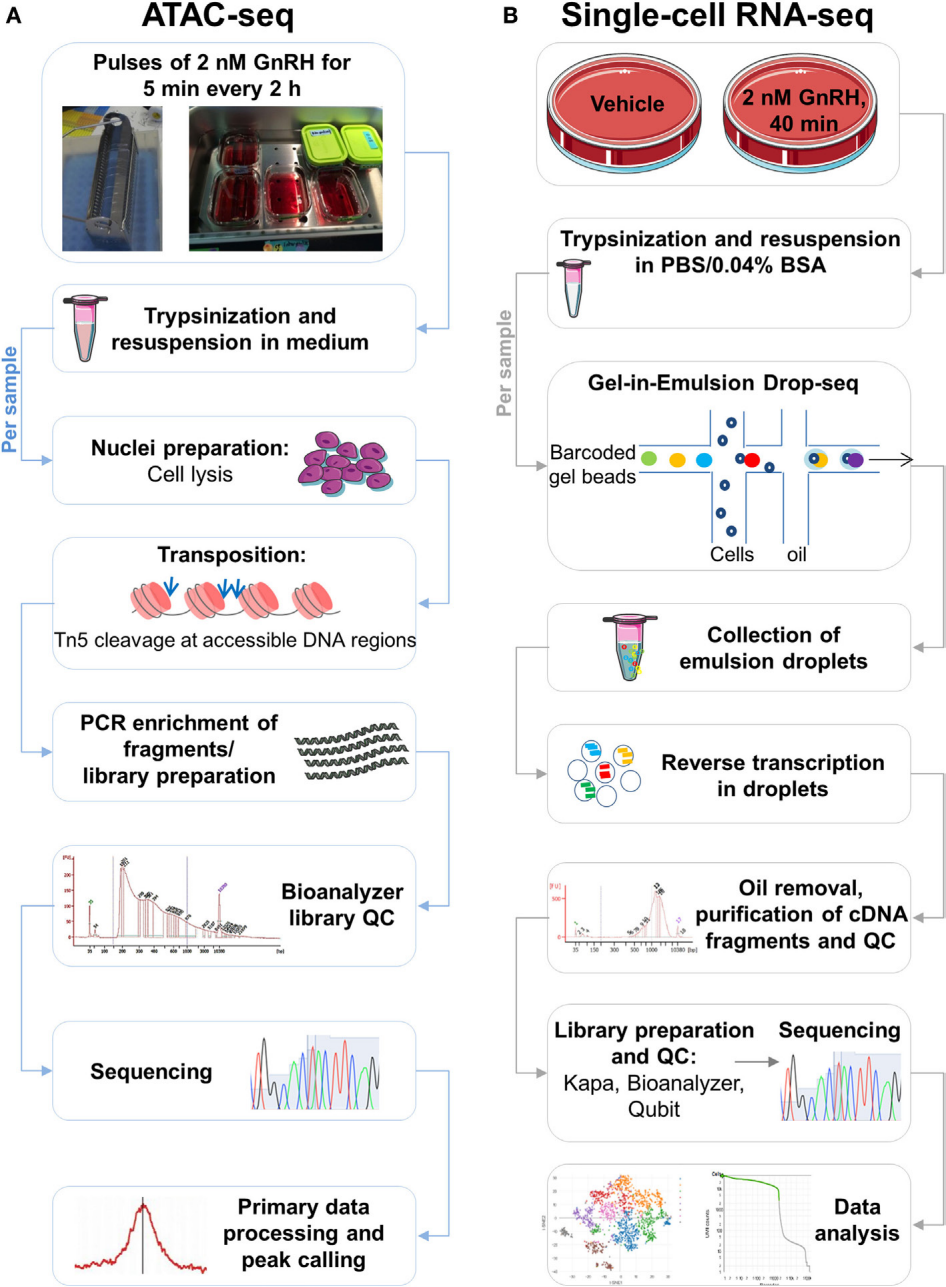
Schematic of the study. **(A)** Assay for transposase-accessible chromatin with high-throughput sequencing (ATAC-seq) workflow. Following treatment with pulses of gonadotropin-releasing hormone (GnRH), LβT2 cells cultured in coverslips placed in racks [see in Ref. (9)] were harvested by trypsinization and resuspended in medium, as indicated. Cell samples were lysed, generating crude nuclei preparations. Next, the transposition reaction was performed in the presence of Tn5 transposase, which cuts and ligates adapters at DNA regions of increased accessibility. The quality of the transposed DNA fragment libraries was assessed using a Bioanalyzer. Libraries were sequenced, mapped to the genome, and processed bioinformatically, and accessible genomic regions were detected (peak calling). **(B)** Single-cell (SC) RNA-seq [gel bead-in-emulsion (GEM) Drop-seq] workflow. Following treatment with GnRH or vehicle, cultured LβT2 cells were harvested by trypsinization and resuspended in a PBS BSA-containing buffer. Cell samples were loaded on a microfluidic chip, combined with reagents and barcoded gel beads to form Gel beads in Emulsion (GEMs), and then mixed with oil. The resulting emulsions were collected and reverse transcribed in SC droplets. Oil was removed, and barcoded cDNA was amplified, purified, and subject to quality control (QC) assessment. Amplified cDNA was incorporated into libraries, which were subsequently subject to QC evaluation using Kapa, Bioanalyzer, and Qubit. Libraries were pooled for sequencing and demultiplexed for subsequent analyses.

An unbiased epigenomic and SC transcriptomic study of LβT2 gonadotrope cells has not been reported. Here, we generated a global mRNA expression profile and a genome-wide atlas of accessible chromatin in GnRH-stimulated LβT2 cells by analyzing RNA-seq data (30) and ATAC-seq data (Figure 1A), respectively. An integrative analysis of the transcriptome and open chromatin data identified key GnRH-regulated genes along with putative *cis*-regulatory elements. We also analyzed cell-to-cell variability of the transcriptome of GnRH-stimulated LβT2 cells and the effects of cell cycle state on this response using GEM Drop-seq (Figure 1B).

## Materials and Methods

### Cell Culture and Treatment

Gonadotropin-releasing hormone was purchased from Bachem (Torrance, CA, USA). LβT2 cells were obtained from Dr. Pamela Mellon (University of California, San Diego, CA, USA). Cells were cultured at 37°*C* in DMEM (Mediatech, Herndon, VA, USA) supplemented with 10% fetal bovine serum (FBS; Gemini, Calabasas, CA, USA) in a humidified air atmosphere of 5% CO_2_. Cells were frozen in freezing medium containing 70% DMEM, 20% FBS, and 10% DMSO (Sigma) and maintained in liquid nitrogen. Cell line authentication was achieved by comparing our cells with an early passage aliquot of LβT2 cells provided by Dr. Mellon and used as a standard reference (Idexx Bioresearch, Columbia, MO, USA). Our results confirmed that our LβT2 cells were *Mycoplasma* free, were of mouse origin, and had similar markers as the original cell line aliquot.

Protocols for GnRH stimulation were selected to provide well-characterized response patterns. Hence, early gene responses in LβT2 cells are sensitive to GnRH concentration, while Fshb transcript levels respond to variations in both GnRH concentration and pulse patterns (9, 13). Because GnRH stimulation of LβT2 cells was previously shown to induce an early gene program, whose transcripts are upregulated within an hour of GnRH exposure (12, 13), a 40- to 45-min GnRH treatment was used in this study to analyze the transcriptome response to GnRH. For the analysis of chromatin accessibility by ATAC-seq, a pattern of pulsatile GnRH exposure was employed to capture the chromatin state that accompanies maximal *Fshb* induction (9, 18).

### Bulk RNA-seq Assay

The generation of the RNA-seq data set that we analyze in this study was previously described (30). Briefly, LβT2 cells were serum starved overnight and stimulated with either 5 nm GnRH or vehicle for 45 min. Each group consisted of four independent replicates. Total RNA (2.5 µg) from each replicate was sequenced at the Mount Sinai Genomics Core Facility using an Illumina platform (Illumina, Inc., San Diego, CA, USA) and a HiSeq 2000 sequencing system (100-nucleotide length, single read type, multiplexing three samples per lane). The RNA-Seq data are deposited in GEO (GSE42120).

### Assay for Transposase-Accessible Chromatin with High-Throughput Sequencing

Assay for transposase-accessible chromatin with high-throughput sequencing was performed as previously described (25) on two replicate samples of LβT2 cells treated with 2 nM GnRH pulses every 2 h for a duration of 6 h and 45 min (4 pulses in total; cells harvested 45 min after last pulse) in a high-throughput GnRH pulse system (9). Briefly, 4,500 cells were washed with cold PBS at 4°C and lysed for 10 min at 4°C. Cell pellets were resuspended in the transposase reaction mix [5 µl 2× TD buffer, 0.5 µl transposase (Illumina) and 4.5 µl nuclease-free water] and incubated at 37°C for 30 min. DNA from the transposase reaction was purified with a DNA Clean & Concentrator-5 Kit (Zymo Research). PCR amplification was performed using Nextera PCR primers. The optimal number of cycles was determined *via* quantitative real-time PCR (qPCR) to stop the amplification before saturation. Libraries were purified with AMPure beads and then quantified using a KAPA Library Quantification Kit (Kapa Biosystems) and High-Sensitivity DNA Bionalyzer kit (Agilent) and sequenced on an Illumina HiSeq 2500 to >164M reads with 50 bp read length, paired-end. The ATAC-seq data are deposited in GEO (GSE102480).

### GEM Drop-seq Assay

Gel bead-in-emulsion Drop-seq was performed as described [10× Genomics, Pleasanton, CA, USA (29)]. Briefly, LβT2 cells were seeded at 350,000 cells per well in 12-well plates for 48 h and treated on day 3 with either 2 nM GnRH or vehicle for 40 min. Cells were then trypsinized and resuspended in medium before being washed and resuspended in 1× PBS/0.04% BSA. Following filtration of the cell suspension, cells were counted on a Countess instrument, and viability was assessed to be above 90% using Trypan Blue. Final concentration was set at 1,000 cells/μl in 1× PBS/0.04% BSA. As a starting point, ~8,000 cells from each sample were loaded into the fluidics chip. Reverse transcription was performed in the emulsion, and cDNA was amplified for 12 cycles before library construction. Quality control (QC) and quantification of the amplified cDNA were assessed using the High-Sensitivity DNA Bioanalyzer kit. Library quality control and quantification were evaluated. The SC data set is deposited in GEO (GSE102480).

### Quantification and QC of RNA and Libraries

RNA concentrations were determined with Quant-iT RiboGreen RNA reagent (Invitrogen, Carlsbad, CA, USA) using a fluorescence microplate reader (SpectraMax M3, Molecular Devices, Sunnyvale, CA, USA). RNA quality was assessed by determining the RNA Integrity Number using Bioanalyzer.

Library QC and quantification were assessed using Nanodrop, Qubit (fluorometric quantitation, ThermoFisher Scientific), Kapa (quantification, Kapa Biosystems), High-Sensitivity DNA Bioanalyzer kit (Agilent), and qPCR of selected genes.

### Quantitative Real-time PCR

Following total RNA isolation, 1 µg of RNA was reverse transcribed with the Affinity Script reverse-transcriptase (Agilent, Santa Clara, CA, USA). Next, samples were diluted 1:20 in molecular biology grade H_2_O (Cellgro, Manassas, VA, USA). SYBR Green qPCR assays were performed (40 cycles) in an ABI Prism 7900HT thermal cycler (Applied Biosystems, Foster City, CA, USA) using 5 µl of cDNA template and 5 µl of master mix containing the specific primers for the targeted gene, Platinum^®^Taq DNA polymerase, and the required qPCR buffer, following the manufacturer’s recommendations. Three technical qPCR replicates were run for each biological replicate. Results were exported as cycle threshold (Ct) values, and Ct values of target genes were normalized to that of *Rps11* in subsequent analysis. Data were expressed as arbitrary units by using the formula, *E* = 2,500 × 1.93(^rps11 CT value − gene of interest CT value)^, where *E* is the expression level in arbitrary units. Primer sequences were as previously described (9, 12).

### Bulk RNA-seq Data Analysis

The RNA-seq data generated about 36–45 million reads per sample. The RNA-seq reads were aligned using STAR (31) v2.5.1b with the mouse genome (GRCm38 assembly) and gene annotations (release M8, Ensembl version 83) downloaded from the https://www.gencodegenes.org/web site. 91–93% of the reads were uniquely mapped to the mouse transcriptome. The matrix counts of gene expression for all eight samples were computed by featureCounts v1.5.0-p1 (32). Differentially expressed genes (5% FDR and at least 2 log2 fold change) were identified using the voom method (33) in the Bioconductor (34) package Limma (35). When comparing the bulk RNA-seq analysis with SC RNA-seq or ATAC-seq data, the transcripts per million (TPM) computed by RSEM (36) was used for the comparison.

### ATAC-seq Data Analysis

Primary data analysis involved read mapping *via* bowtie2 (37) to GRCm38 (mm10), followed by duplicate read removal and peak calling *via* MACS2 (38) with parameters “-g dm –nomodel –shiftsize 100 extsize 200.” For examining correspondence between chromatin accessibility and gene expression (Figure 4A), all peaks were first annotated to the nearest gene, and the most significant peak score for each gene was selected.

### SC RNA-seq Data Analysis

Single-cell RNA-seq data were processed using the Cell Ranger pipeline v1.3, which provides a data matrix of expression for all genes and all cells. Differentially expressed genes were analyzed using the sSeq method (41), as implemented in the R package cellrangerRkit v1.1. The cell phase computation for the single cells follows the ideas described in the Supplementary Material of the study by Macosko et al. (28) with our own customized R script implementation. A schematic of the cell phase score computation is described in Figure 6A. The t-SNE analysis (42, 43) was performed using the implementation from the Cell Ranger pipeline.

## Results

### Transcriptome Profiling of LβT2 Cells

**Figure 2:**
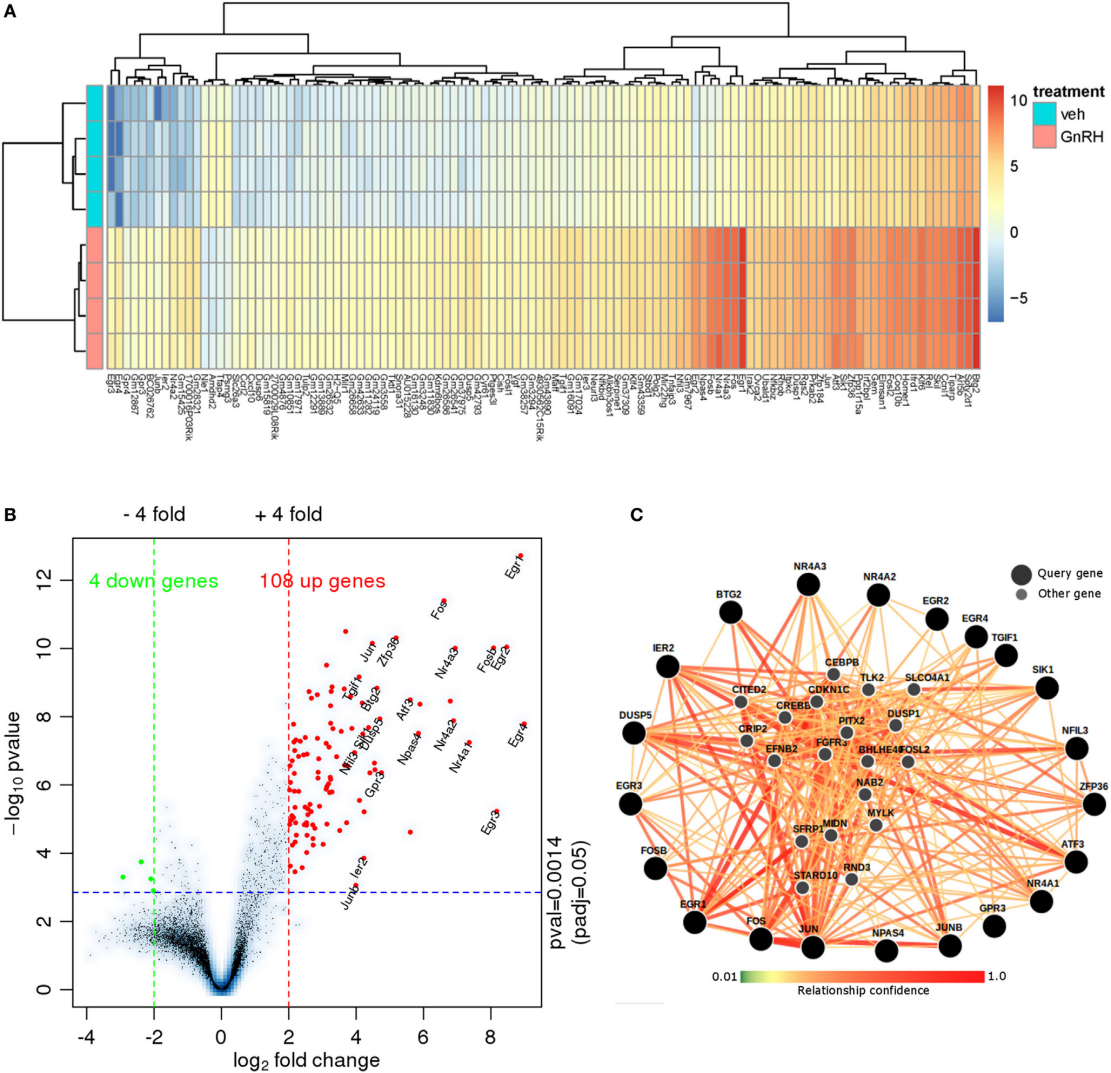
RNA-seq data analysis. **(A)** Heat map of RNA-seq expression data showing the genes that were differentially regulated following treatment with 5 nM gonadotropin-releasing hormone (GnRH) for 45 min. Gene expression is shown in normalized log2 counts per million. Differentially expressed genes were selected based on a fourfold change and FDR < 0.05. **(B)** Volcano plot with the log2 fold changes in gene expression after GnRH treatment on the x-axis and the statistical significance (−log10 *p* value) on the y-axis. The gene symbols of 21 annotated genes with at least a 16-fold expression change are displayed. **(C)** Functional network analysis of the top 21 genes using (Genome-scale Integrated Analysis of gene Networks in Tissues) (39). Nodes related to those 21 genes are shown (smaller markers) in this human hypophysis (pituitary) data-driven Bayesian functional network.

To characterize the transcriptome response to GnRH in LβT2 cells for comparison with the chromatin accessibility and SC studies described below, we analyzed an RNA-seq experiment in which LβT2 cells were exposed to either GnRH (5 nM) or vehicle for 45 min [(30); n = 4 for each group]. We identified 112 differentially expressed genes relative to the control (>4-fold at FDR < 0.05, see Figure 2A; Figure S1 in Supplementary Material and Table S1 in Supplementary Material), including a large number of early gene transcripts that are known to be regulated by GnRH in gonadotropes, such as Egr1, Fos (c-Fos), and Jun (c-Jun) (12, 45, 46). As is evident from the asymmetrical expression change versus significance volcano plot (Figure 2B), the majority of regulated transcripts were upregulated at this time point. The plot highlights the most highly upregulated genes, which include Jun, Fos, Fosb, Egr1, Egr2, Egr3, Egr4, Nr4a3, and Nr4a1.

We investigated the functional relationships of the 21 highest fold-change annotated GnRH-regulated genes in the context of a human pituitary gene network using Genome-scale Integrated Analysis of gene Networks in Tissues [GIANT; Figure 2C; (39)]. GIANT uses a massive public data compendium to infer tissue-specific data-driven functional gene–gene relationships. The output of a GIANT analysis is a graph indicating the strength of the functional relationship of each pair of the input genes in the pituitary, as well as the inference of additional highly related genes. The edges connecting the nodes indicate the statistical strength of the evidence for that relationship in the human pituitary. Notably, the highest regulated genes form highly interrelated subgroups (e.g., *Jun*/*Fos*/*Ier2*), and many highly related inferred genes are regulated by GnRH (e.g., *Dusp1, Nab2*) or known to be central to key gonadotropin developmental or regulatory processes [e.g., *Pitx2*; Table S2 in Supplementary Material; (12, 14, 47–50)]. Included among the statistically significant gene enrichment set were MAPK signaling and SMAD protein signaling (Table S3 in Supplementary Material). Overall, this analysis reveals a coordinated gene program activated by short-term GnRH exposure.

### Genome-Wide Mapping of Chromatin Accessibility in LβT2 Cells

**Figure 3:**
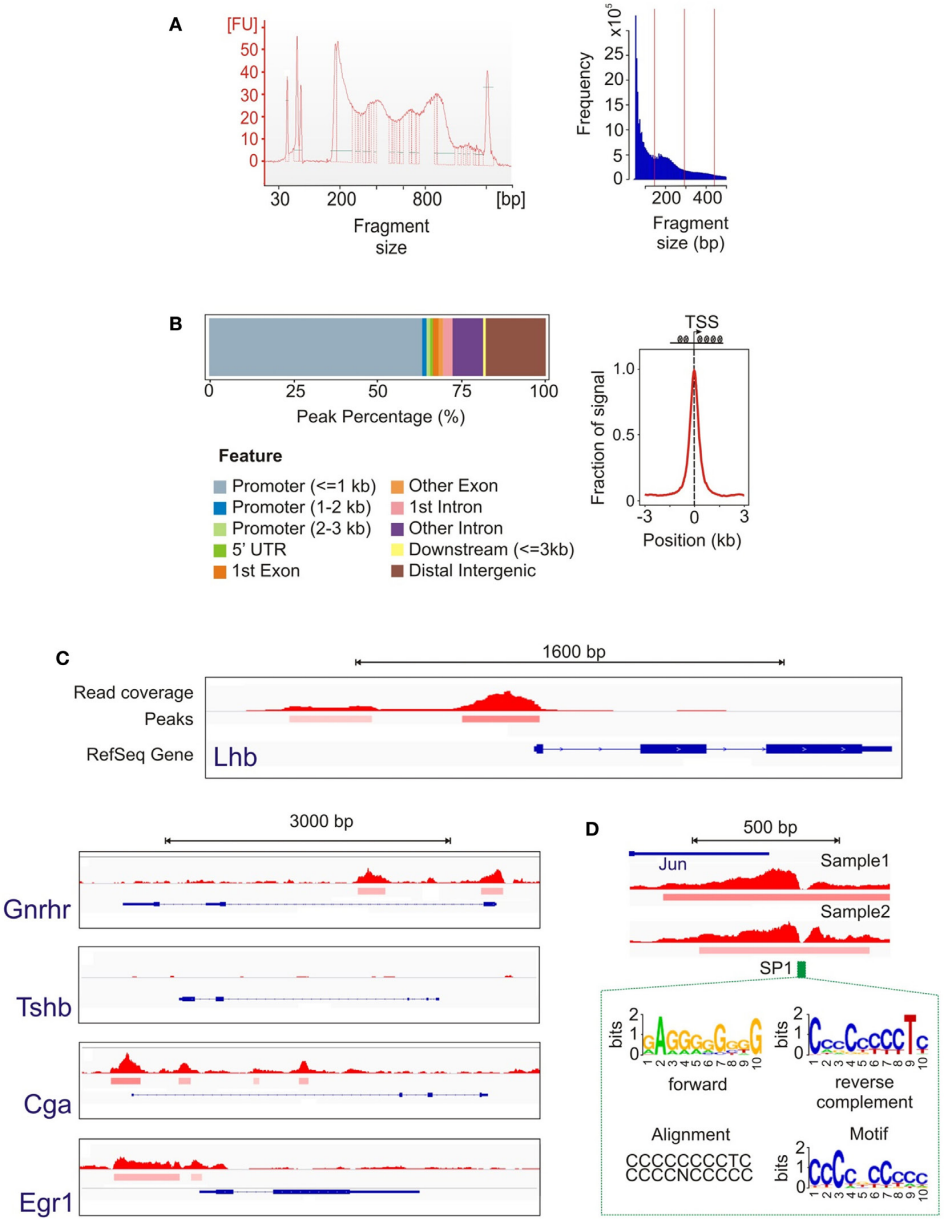
Assay for transposase-accessible chromatin with high-throughput sequencing (ATAC-seq) data analysis. **(A)** Library and sequence fragment size distribution showing a periodicity characteristic of the effect of nucleosome binding. **(B)** Position of open chromatin peaks with respect to genomic features shows their genome-wide distribution are concentrated around the active transcription start sites (TSS), indicating that TSS are enriched for open chromatin. **(C)** Representative loci of genes showing the open chromatin signal. The top track represents the read coverage (density of sequence reads) at each location. The middle peak track indicates genomic regions that achieve statistical significance for chromatin accessibility. The bottom track shows the RefSeq gene annotation (intron/exon locations, transcription direction, etc.). As expected, accessible chromatin segments were detected around the promoter regions for *Lhb, Gnrhr, Cga*, and *Egr1*, but not for *Tshb*. **(D)** Example of transcription factor binding identification based on the footprint analysis of the open chromatin signal. The notch in the open chromatin region corresponds to the location of a consensus SP1 site. The HOMER analysis suite utilized (40) calculates a data-driven motif model from our ATAC-seq peak data, shown in forward and reverse configurations, and then associates this motif with known motifs. In this example, the known SP1 consensus motif is nearly identical to the data-driven motif.

To map open chromatin regions, we carried out ATAC-seq in LβT2 cells treated with GnRH (Figure 1A). ATAC-seq uses the hyperactive Tn5 transposase, loaded with adapters for high-throughput DNA sequencing, to integrate into regions of accessible chromatin. The resulting DNA fragments, generated from locations of open chromatin, are amplified, sequenced, and computationally mapped to the genome to obtain a genome-wide accessibility landscape (24, 25). The ATAC-seq libraries and the sequence data showed a characteristic ~200 bp size distribution periodicity (Figure 3A), reflecting individual nucleosome occupancy patterns and confirming specific transposase activity and assay accuracy. Primary data analysis (see Materials and Methods) identified more than 58,000 statistically significant regions of open chromatin (peaks). The open chromatin map was found to be reproducible across independent samples and libraries. We note that, while determining whether chromatin accessibility changes with GnRH exposure is an important question, the present study was intended only to provide a baseline analysis of open chromatin structure. Performing a reliable comparative analysis of changes in specific regions with GnRH stimulation would require assaying a large number of samples, which was not feasible for this investigation.

We examined the 2,000 most prominent open chromatin regions (showing the highest peak scores; see Materials and Methods) with respect to their location relative to annotated genomic features. Approximately 75% of these peaks were located in immediate gene promoters or introns, which is consistent with open chromatin in the proximal regions of transcriptionally active genes (Figure 3B). Focusing on the sequence fragments in genomic areas flanking the transcription start sites (TSS, −3 to 3 kb), we observed a distribution highly preferential to the regions in close vicinity to the TSS (Figure 3B). We next examined chromatin state in the proximal promoter regions of several key genes (Figure 3C). While open chromatin peaks were detected at genes that are either constitutively expressed in LβT2 cells (*Gnrhr*) and/or regulated by GnRH (*Lhb, Cga*, and *Egr1*), chromatin was closed at the *Tshb* gene that is not expressed in gonadotropes. To gain insight into the overall transcriptional regulatory state of these cells, we used the HOMER tool to determine TF binding motifs showing enrichment among all open chromatin regions (40). The enrichment results show extremely high statistical significance (see Table S4 in Supplementary Material), reflecting the high power of this global analysis. Finding highly enriched global binding motifs for the TFs Smad2 and Six6, which have been implicated in *Fshb* gene expression (51, 52), suggests that this analysis is likely to have generated useful leads for further study. In these high-resolution ATAC-seq data, we were also able to observe TF footprints, which create a notch in a region of otherwise open chromatin due to the presence of a bound TF. As an example, Figure 3D shows a notch detected in the otherwise open *Jun* promoter that precisely matches an SP1 consensus site. This finding is consistent with the known role of SP1 in mediating induction of this gene (53).

**Figure 4:**
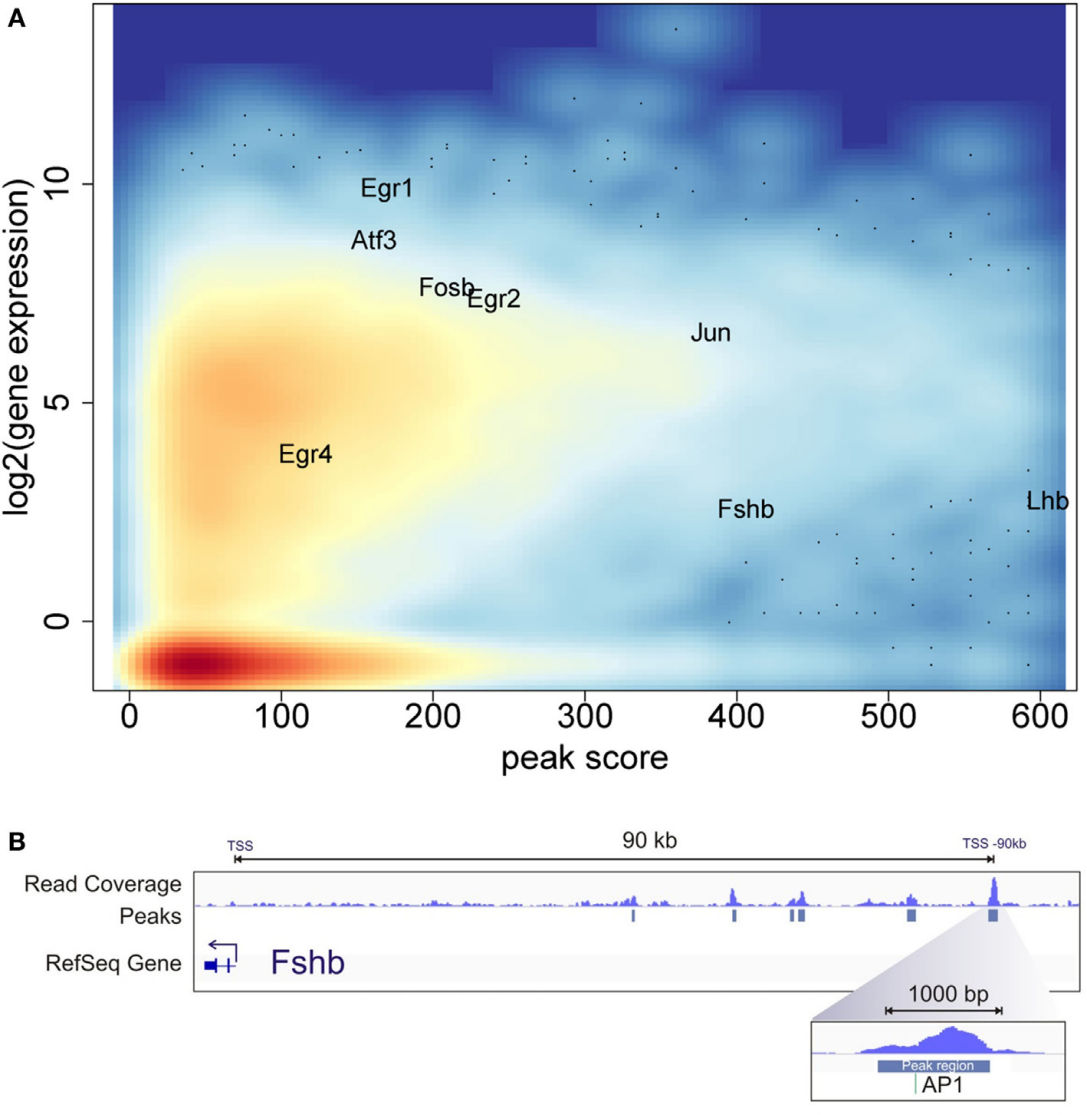
Chromatin accessibility and gene expression landscape. **(A)** Average log2 gene expression for each gene from the RNA-seq experiment compared to the highest assay for transposase-accessible chromatin with high-throughput sequencing (ATAC-seq) peak score associated with the same gene. Color intensity indicates density of gene population, with red representing the highest density and light blue the lowest. **(B)** ATAC-seq and computational analysis of the upstream region of the *Fshb* gene. The top track shows read coverage. The middle track shows the location of significant peak regions. The bottom track indicates the *Fshb* transcription start sites. The direction of *Fshb* transcription is to the left in this diagram. The most distal open chromatin region is the strongest peak detected near *Fshb* and encompasses a consensus AP1 site.

To examine the relationship of the epigenetic landscape and the global pattern of mRNA expression, we compared the ATAC-seq and RNA-seq data. While the GnRH treatment conditions used for ATAC-seq versus RNA-seq experiments differed, they were both compatible with early gene induction. Indeed, previous analysis of temporal responses of the early genes *Fos* and *Egr1* in response to GnRH pulse stimulation demonstrated that they are highly expressed within 40 min after the fourth pulse (9). In this analysis, we identified the open chromatin peak showing the highest ATAC-seq peak score associated with each annotated gene. This estimate of chromatin accessibility for each gene was plotted against its level of expression following GnRH treatment using the RNA-seq data (Figure 4A). The results show a general pattern of increased chromatin accessibility for expressed genes. Genes found to be highly induced by GnRH (e.g., *Egr1, Egr2, Egr4, Jun, Fosb, Atf3*) tend to show high chromatin accessibility.

The gonadotropin subunit genes *Lhb* and *Fshb* showed very high levels of chromatin accessibility at specific locations. The *Lhb* area of high accessibility, shown in Figure 3C, is centered around the proximal promoter. In contrast, *Fshb* does not show statistically significant open chromatin in the region of the proximal promoter. Notably, several significant open chromatin peaks were found between −40 and −90 kb upstream of the *Fshb* TSS. The segment of highest chromatin accessibility, at −90 kb, contains a binding motif for AP1, a known *Fshb* regulator [Figure 4B; (14)]. This pattern raises the possibility that this region may represent a distal *Fshb* enhancer.

### SC Transcriptome Analysis of LβT2 Cells and Characterization of Early Gene Response to GnRH

To investigate cell-to-cell variability in gene expression in the gonadotrope response to GnRH, we performed a SC transcriptome analysis of LβT2 cells exposed to either GnRH or vehicle for 40 min using GEM Drop-seq (see Figure 1B). This assay measures the entire transcriptome in thousands of individual cells from each sample. Specifically, we sought to assess cell-to-cell heterogeneity with respect to the individual cell response to GnRH.

**Figure 5:**
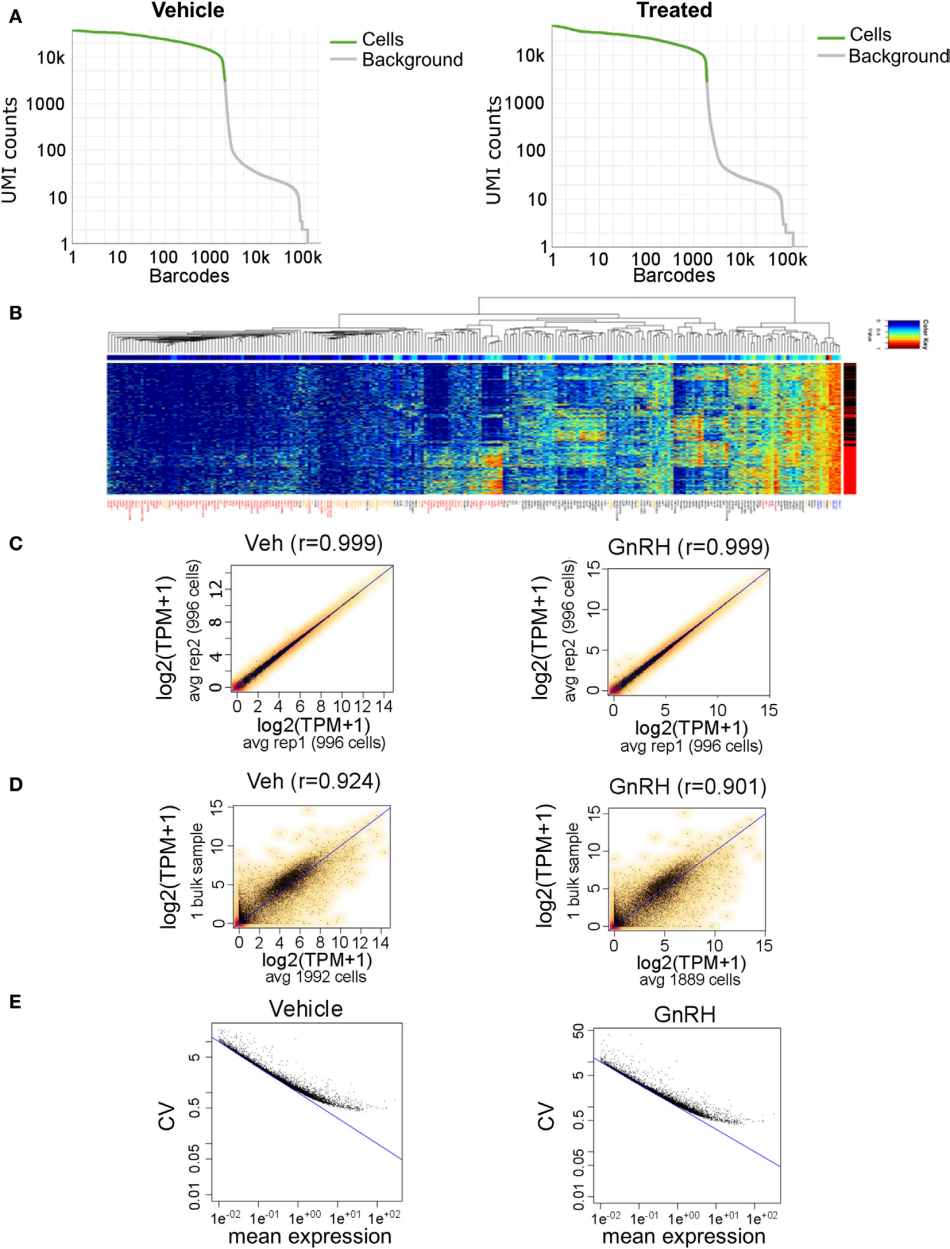
Gel bead-in-emulsion Drop-seq data. Analysis of 3,881 LβT2 cells (1,992 vehicle-treated and 1,889 GnRH-treated cells for 40 min) having a median of 4,027 distinct genes quantified per cell from 380 million reads. **(A)** Drop-off of expression in unique molecular identifiers (UMIs) counts for each cell (barcode), demonstrating even coverage. **(B)** Heat map of selected genes in all 3,881 cells. Each horizontal line is one cell, with red and black on the right side indicating gonadotropin-releasing hormone (GnRH) and vehicle treatment, respectively. Gene symbols are color coded as follows: red, genes that are differentially regulated by GnRH; black, genes that show high cell-to-cell variability in gene expression; yellow, genes that have been involved in gonadotrope gene regulation based on the literature; and blue, housekeeping genes. **(C)** Scatter plots of transcripts per million (TPM) averages obtained for each gene from randomly dividing the population of cells in each sample into two subgroups: vehicle-treated subgroups (left) and GnRH-treated subgroups (right). **(D)** Scatter plots of TPM averaged across all SC and TPM from the bulk RNA-seq data, in either vehicle- or GnRH-treated cells. **(E)** Scatter plots of coefficient of variation versus mean expression level (average number of reads per gene) across all vehicle-treated cells (left) and GnRH-treated cells (right). The blue line denotes the Poisson technical noise, while the black dots signify total variation, which includes cell-to-cell biological variation.

We started with ~8,000 LβT2 cells in each sample. The resulting SC RNA-seq libraries all exhibited the expected electropherogram traces on the Agilent Bioanalyzer High Sensitivity Chip, thus passing the QC assessment. In addition, qPCR assays of several early response genes in the library provided further evidence that our SC RNA-seq libraries were suitable for the detection of individual gene expression and regulation (data not shown). We sequenced to a depth of ~300 million reads in the SC libraries from each sample (vehicle- and GnRH-treated LβT2 cells). We obtained good sequence data for 1,992 vehicle-treated and 1,889 GnRH-treated cells, with ~98,000 mean reads/cell and >4,000 median genes/cell above detection threshold. Analysis of the GEM Drop-seq data demonstrated even coverage, with the slow drop-off of expression/cell in the top nearly 2,000 cells being similar in untreated versus GnRH-treated cells (Figure 5A). These features are indicative of a high-quality SC transcriptome data set.

A heat map of selected genes expressed in all 3,881 analyzed cells revealed a global pattern of differential expression between GnRH-treated and vehicle-treated cells (Figure 5B). We identified 95 differentially expressed genes that included known GnRH-regulated immediate-early genes such as *Egr1, Fos, Fosb, Jun, Btg2, Junb*, and *Nr4a1* (Table S5 in Supplementary Material). Notably, we observed high cell-to-cell heterogeneity in response to GnRH, and several GnRH-treated cells even exhibited a gene expression pattern similar to that of untreated cells (Figure 5B; Figure S2 in Supplementary Material). Comparison of SC gene expression measurements averaged across two randomly selected subgroups of 996 cells highlighted the high reproducibility and consistency of the data, as the correlation coefficient was >0.99 in either vehicle- or GnRH-treated cells (Figure 5C). To further evaluate the performance of the digital transcriptome, we compared Drop-seq gene expression measurements averaged across all SC with the bulk RNA-seq measurements analyzed above, in either vehicle- or GnRH-treated cells. Despite the SC and bulk sequencing data sets coming from different experiments, the aggregated Drop-seq data showed high correlation with the bulk RNA-seq data (correlation coefficient >0.90; Figure 5D). The high cell-to-cell variation in the response to GnRH and in gene expression levels results from a combination of technical measurement variation and true cell-to-cell biological variation. To gain a sense of the degree of cell expression measurement resulting from technical noise, we modeled the technical measurement variation by a Poisson distribution [(54); Figure 5E]. Notably, at high levels of expression, the contribution of technical noise is relatively small and supports the presence of high levels of true biological cell-to-cell expression variation in these cells.

### Analysis of Cell Cycle Dependence of the SC Transcriptome of GnRH-Stimulated LβT2 Cells

One limitation in many studies of response to GnRH performed by our group and others in LβT2 cells is that the effects of cell division on GnRH-induced gene regulation are not controlled. LβT2 cultures comprise asynchronously dividing cells. A SC transcriptome experiment provides the possibility of identifying the cell cycle stage of each individual cell and determining whether cell cycle stage influences the response to GnRH. Previous studies in yeast indicate that cell cycle has global effects on protein and RNA synthesis, thus affecting the transcriptional activity (55–57). RNA levels are controlled by transcriptional bursting that can vary during different cell cycle stages (58).

**Figure 6:**
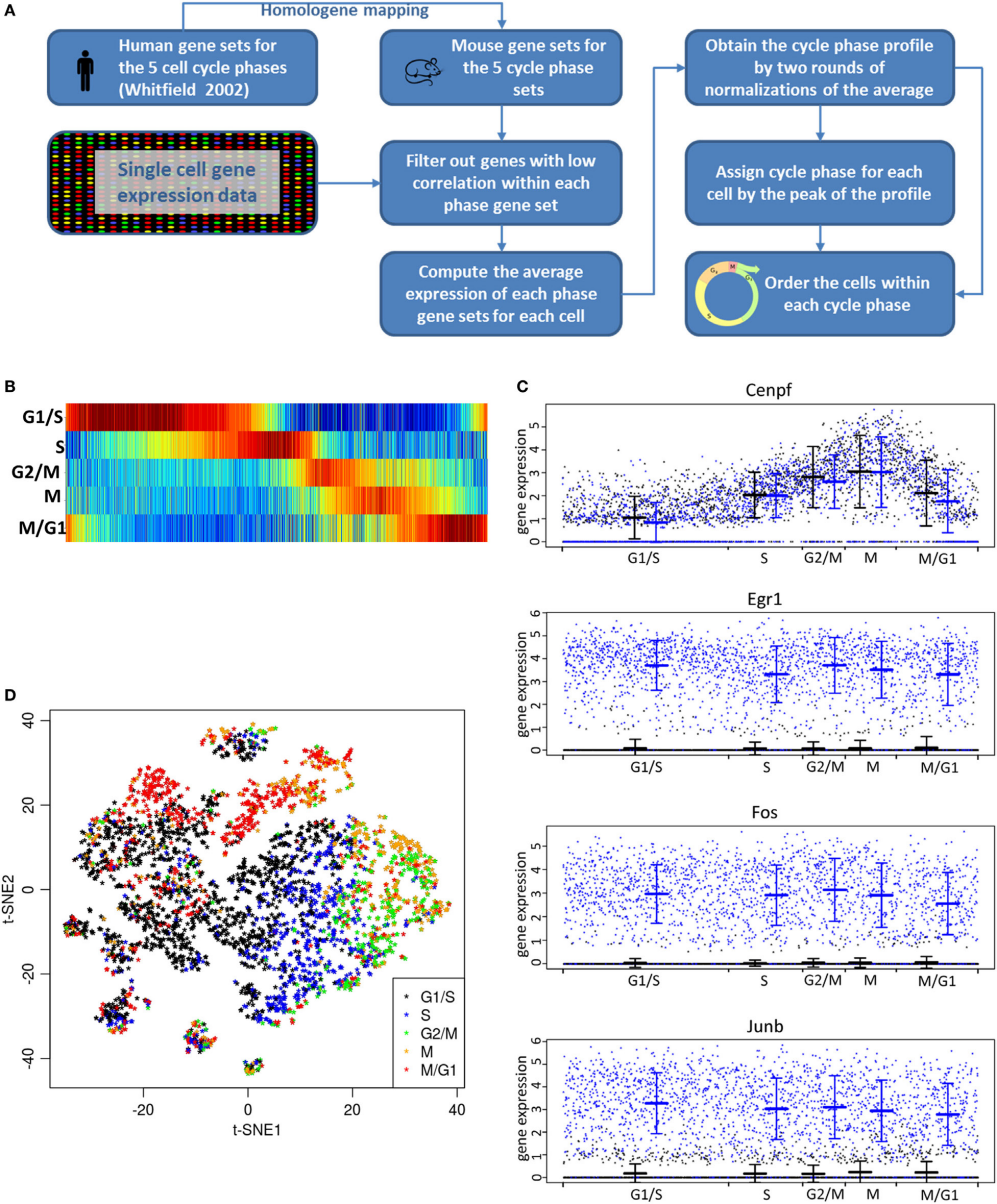
Analysis of effects of cell cycle. **(A)** Flowchart diagram of the cell cycle analysis method. The score computation is based on the Supplemental Material from the study by Macosko et al. (28) with the cell cycle genes taken from their Table S2 in Supplementary Material mapped to mouse gene using Homologene. The gene sets for the five cell cycle phases (G1/2, S, G2/M, M, and M/G1) were determined by comparing cells that were arrested at different cell cycle phases (44). **(B)** Each of the 3,881 (GnRH- and vehicle-treated) cells on the x-axis is aligned by the cell cycle progression. Each vertical line represents one cell with the color code indicating low (dark blue) to high (dark red) score assignment to the cell cycle phase. All cells are ranked by cell cycle progression according to the steps depicted in **(A)**. The five cell cycle phases are indicated by the labels on the horizontal lines. **(C)** Single-cell expression of individual transcripts in relationship to cell cycle phase. Shown is the expression [log2 (TPM + 1)] of a gene associated with the mitotic (M) phase of cell cycle (*Cenpf*) and of GnRH-regulated genes (*Egr1, Fos*, and *Junb*). X-axis indicates cell cycle progression, as derived from **(B)**. The mean and SD for vehicle- (in black) and GnRH-treated cells (in blue) are shown at each cell cycle phase. Note that the expression level of any gene in these cells at any point in the cell cycle can be accurately determined without experimental cell cycle synchronization. **(D)** t-SNE plot of all 3,881 cells based on global expression of all genes (see Materials and Methods). The color of each cell indicates the cell cycle phase as determined in **(B)**. The fact that cells are clustered according to the cell cycle phase indicates that the primary cause of gene expression change is the cell cycle phase change.

We examined cell cycle state of LβT2 cells as well as the influence of cell cycle phase on gene expression. Comparison of each cell’s average expression with gene sets known to be enriched in one of the five cycle phases [G1/S, S, G2/M, M, and M/G1; Table S6 in Supplementary Material; for reference, see Ref. (44)] enabled alignment of each cell by the cell cycle stage (Figures 6A,B). Consistent with this alignment, a gene associated with the M cycle phase (*Cenpf*) exhibited the highest expression at the M phase and high expression at the G2/M phase (Figure 6C). In contrast, *Cenpf* was poorly expressed at G1/S and moderately expressed at S and M/G1. Likewise, the expression levels of other genes known to be associated with a specific phase of the cell cycle (e.g., *Pcna* with G1/S and S, *Top2a* with both S and G2/M) were highest at that cell cycle phase (Figure S3 in Supplementary Material). On the other hand, immediate-early genes induced by GnRH (e.g., *Egr1, Fos, Junb*) and other GnRH-regulated genes (e.g., *Gdf9*) showed no significant expression change with cell cycle phase (Figure 6C). The overall cell distribution with respect to cell cycle phase was comparable in GnRH-treated cells versus control (vehicle-treated cells) cells (data not shown). Additional analysis showed that the five cycle phases were represented throughout all analyzed cells, with individual cells partly forming clusters based on their cell cycle phase (Figure 6D).

## Discussion

In this study, we analyzed the global transcriptional, epigenetic, and single-cell transcriptional landscapes of the LβT2 gonadotrope cell line using RNA-seq data, ATAC-seq data and GEM Drop-seq data, respectively. To our knowledge, this represents the first genome-wide epigenetic characterization and the first SC transcriptome study performed in any gonadotrope experimental system. Our results provide insight into the global transcriptional regulatory processes of these cells and provide data sets and hypotheses to guide further work in this field.

A recent characterization of epigenetics in gonadotrope models reported CpG methylation, DNAse hypersensitivity, and histone modification at regions of several specific genes in several cell lines, including LβT2 cells (59). Their finding of open chromatin correlating with expression at the specific genes investigated corresponds with our genome-wide characterization of this relationship. The global ATAC-seq epigenetic approach that we have pursued opens the avenue to entirely unexpected discovery, such as the putative new *Fshb* enhancer identified as highly open chromatin (see Figure 4B). Further study is needed to evaluate the functional role of this novel putative enhancer.

Analysis of SC transcriptome in vehicle- and GnRH-treated cells demonstrates, for the first time, that there is no influence of cell cycle stage on the gene response to GnRH. The SC variability observed at the level of basal gene expression and gene induction by GnRH is high and largely not explained by the technical variation to which SC transcriptome analysis is prone (60). High levels of SC expression and gene induction variation in LβT2 cells could be anticipated based on previous studies of response variation and noise scale of selected transcripts (61, 62). The cause of this high level of variation in expression and response to GnRH in LβT2 cells and whether it accurately models the expression and response patterns of the intact mouse gonadotrope are unknown. While recapitulating many properties of a mature gonadotrope, LβT2 cells are a transformed cell line generated by tumorigenesis. It is conceivable that the heterogeneity of these cells has been augmented by the process of transformation (63, 64). Cell-to-cell variation in expression and response to stimulation can also result from normal biological variation and stochastic mechanisms (65). Thus, alternatively, this line may faithfully reflect SC expression and response variation and represent an accurate model of the behavior of the primary gonadotrope.

Individual gonadotropes largely function as a SC processor in controlling the reproductive axis and understanding the role of cell-to-cell variation in the engineering of this system is a relevant question to approach through SC biology. Evaluation of chromatin accessibility variation at the SC level [SC ATAC-seq (66, 67)] is also an interesting area for future investigation. The generation of high-quality global ATAC-seq chromatin accessibility and SC GEM Drop-seq transcriptome data should provide a useful resource for the research community.

## Author Contributions

FR-Z and MF designed and performed research, analyzed, and interpreted data; YG, EZ, GN, ST, and HW contributed analytic tools and analyzed data; VN performed research and analyzed data; HP interpreted data and drafted the work; JT analyzed and interpreted data; SC conceived research, analyzed data, and drafted the work. All authors drafted or revised the work critically and approved the final version to be submitted.

## Conflict of Interest Statement

The authors declare that the research was conducted in the absence of any commercial or financial relationships that could be construed as a potential conflict of interest.

## Abbreviations

ATAC-seq, assay for transposase-accessible chromatin with high-throughput sequencing; FSH, follicle-stimulating hormone; GIANT, genome-scale integrated analysis of gene networks in tissues; GEM, gel bead-in-emulsion; GnRH, gonadotropin-releasing hormone; LH, luteinizing hormone; SC, single cell; TF, transcription factor; TSS, transcription start site.

## References

[1] Marshall JC Dalkin AC Haisenleder DJ Paul SJ Ortolano GA Kelch RP. Gonadotropin-releasing hormone pulses: regulators of gonadotropin synthesis and ovulatory cycles. Recent Prog Horm Res (1991) 47:155–87; discussion 188–59. 1745819 10.1016/b978-0-12-571147-0.50009-3

[2] Haisenleder DJ Dalkin AC Ortolano GA Marshall JC Shupnik MA. A pul-satile gonadotropin-releasing hormone stimulus is required to increase transcription of the gonadotropin subunit genes: evidence for differential regulation of transcription by pulse frequency in vivo. Endocrinology (1991) 128(1):509–17. 10.1210/endo-128-1-509 1702704

[3] Choi SG Jia J Pfeffer RL Sealfon SC. G proteins and autocrine signaling differentially regulate gonadotropin subunit expression in pituitary gonadotrope. J Biol Chem (2012) 287(25):21550–60. 10.1074/jbc.M112.348607 22549790 PMC3375576

[4] Alarid ET Windle JJ Whyte DB Mellon PL. Immortalization of pituitary cells at discrete stages of development by directed oncogenesis in transgenic mice. Development (1996) 122(10):3319–29. 8898243 10.1242/dev.122.10.3319

[5] Thomas P Mellon PL Turgeon J Waring DW. The L beta T2 clonal gonadotrope: a model for single cell studies of endocrine cell secretion. Endocrinology (1996) 137(7):2979–89. 10.1210/endo.137.7.8770922 8770922

[6] Turgeon JL Kimura Y Waring DW Mellon PL. Steroid and pulsatile gonadotropin-releasing hormone (GnRH) regulation of luteinizing hormone and GnRH receptor in a novel gonadotrope cell line. Mol Endocrinol (1996) 10(4):439–50. 10.1210/mend.10.4.8721988 8721988

[7] Graham KE Nusser KD Low MJ. LbetaT2 gonadotroph cells secrete follicle stimulating hormone (FSH) in response to active A. J Endocrinol (1999) 162(3):R1–5. 10.1677/joe.0.162R001 10467239

[8] Pernasetti F Vasilyev VV Rosenberg SB Bailey JS Huang HJ Miller WL Cell-specific transcriptional regulation of follicle-stimulating hormone-beta by activin and gonadotropin-releasing hormone in the LbetaT2 pituitary gonadotrope cell model. Endocrinology (2001) 142(6):2284–95. 10.1210/endo.142.6.8185 11356674

[9] Stern E Ruf-Zamojski F Zalepa-King L Pincas H Choi SG Peskin CS Modeling and high-throughput experimental data uncover the mechanisms underlying Fshb gene sensitivity to gonadotropin-releasing hormone pulse frequency. J Biol Chem (2017) 292(23):9815–29. 10.1074/jbc.M117.783886 28385888 PMC5465502

[10] Stojilkovic SS Catt KJ Calcium oscillations in anterior pituitary cells. Endocr Rev (1992) 13(2):256–80. 10.1210/edrv-13-2-256 1319898

[11] Bjelobaba I Janjic MM Tavcar JS Kucka M Tomic M Stojilkovic SS. The relationship between basal and regulated Gnrhr expression in rodent pituitary gonadotrophs. Mol Cell Endocrinol (2016) 437:302–11. 10.1016/j.mce.2016.08.040 27569529 PMC6364298

[12] Wurmbach E Yuen T Ebersole BJ Sealfon SC. Gonadotropin-releasing hormone receptor-coupled gene network organization. J Biol Chem (2001) 276(50):47195–201. 10.1074/jbc.M108716200 11581274

[13] Yuen T Wurmbach E Ebersole BJ Ruf F Pfeffer RL Sealfon SC. Coupling of GnRH concentration and the GnRH receptor-activated gene program. Mol Endocrinol (2002) 16(6):1145–53. 10.1210/mend.16.6.0853 12040003

[14] Coss D Jacobs SB Bender CE Mellon PL. A novel AP-1 site is critical for maximal induction of the follicle-stimulating hormone beta gene by gonadotropin-releasing hormone. J Biol Chem (2004) 279(1):152–62. 10.1074/jbc.M304697200 14570911 PMC2930619

[15] Nicol L Faure MO McNeilly JR Fontaine J Taragnat C McNeilly AS. Bone morphogenetic protein-4 interacts with activin and GnRH to modulate gonadotrophin secretion in LbetaT2 gonadotrophs. J Endocrinol (2008) 196(3):497–507. 10.1677/JOE-07-0542 18310445 PMC2262182

[16] Kanasaki H Mutiara S Oride A Purwana IN Miyazaki K. Pulse frequency-dependent gonadotropin gene expression by adenylate cyclase-activating polypeptide 1 in perifused mouse pituitary gonadotroph LbetaT2 cells. Biol Reprod (2009) 81(3):465–72. 10.1095/biolreprod.108.074765 19458315

[17] Ho CC Bernard DJ. Bone morphogenetic protein 2 acts via inhibitor of DNA binding proteins to synergistically regulate follicle-stimulating hormone beta transcription with activin A. Endocrinology (2010) 151(7):3445–53. 10.1210/en.2010-0071 20463050

[18] Choi SG Wang Q Jia J Pincas H Turgeon JL Sealfon SC Growth differentiation factor 9 (GDF9) forms an incoherent feed-forward loop modulating follicle-stimulating hormone beta-subunit (FSHbeta) gene expression. J Biol Chem (2014) 289(23):16164–75. 10.1074/jbc.M113.537696 24778184 PMC4047387

[19] Choi SG Wang Q Jia J Chikina M Pincas H Dolios G Characterization of gonadotrope secretoproteome identifies neurosecretory protein VGF-derived peptide suppression of follicle-stimulating hormone gene expression. J Biol Chem (2016) 291(40):21322–34. 10.1074/jbc.M116.740365 27466366 PMC5076536

[20] Pincas H Choi SG Wang Q Jia J Turgeon JL Sealfon SC. Outside the box signaling: secreted factors modulate GnRH receptor-mediated gonadotropin regulation. Mol Cell Endocrinol (2014) 385(1–2):56–61. 10.1016/j.mce.2013.08.015 23994024 PMC3964483

[21] Ferris HA Walsh HE Stevens J Fallest PC Shupnik MA. Luteinizing hormone beta promoter stimulation by adenylyl cyclase and cooperation with gonadotropin-releasing hormone 1 in transgenic mice and LBetaT2 Cells. Biol Reprod (2007) 77(6):1073–80. 10.1095/biolreprod.107.064139 17699734

[22] Ely HA Mellon PL Coss D. GnRH induces the c-Fos gene via phosphorylation of SRF by the calcium/calmodulin kinase II pathway. Mol Endocrinol (2011) 25(4):669–80. 10.1210/me.2010-0437 21292826 PMC3063085

[23] Fortin J Kumar V Zhou X Wang Y Auwerx J Schoonjans K NR5A2 regulates Lhb and Fshb transcription in gonadotrope-like cells in vitro, but is dispensable for gonadotropin synthesis and fertility in vivo. PLoS One (2013) 8(3):e59058. 10.1371/journal.pone.0059058 23536856 PMC3594184

[24] Buenrostro JD Giresi PG Zaba LC Chang HY Greenleaf WJ. Transposition of native chromatin for fast and sensitive epigenomic profiling of open chromatin, DNA-binding proteins and nucleosome position. Nat Methods (2013) 10(12):1213–8. 10.1038/nmeth.2688 24097267 PMC3959825

[25] Buenrostro JD Wu B Chang HY Greenleaf WJ ATAC-seq: a method for assaying chromatin accessibility genome-wide. Curr Protoc Mol Biol (2015) 109: 21.29.21–9. 10.1002/0471142727.mb2129s109 PMC4374986 25559105

[26] Kowalczyk MS Tirosh I Heckl D Rao TN Dixit A Haas BJ Single-cell RNA-seq reveals changes in cell cycle and differentiation programs upon aging of hematopoietic stem cells. Genome Res (2015) 25(12):1860–72. 10.1101/gr.192237.115 26430063 PMC4665007

[27] Klein AM Mazutis L Akartuna I Tallapragada N Veres A Li V Droplet barcoding for single-cell transcriptomics applied to embryonic stem cells. Cell (2015) 161(5):1187–201. 10.1016/j.cell.2015.04.044 26000487 PMC4441768

[28] Macosko EZ Basu A Satija R Nemesh J Shekhar K Goldman M Highly parallel genome-wide expression profiling of individual cells using nanoliter droplets. Cell (2015) 161(5):1202–14. 10.1016/j.cell.2015.05.002 26000488 PMC4481139

[29] Zheng GX Terry JM Belgrader P Ryvkin P Bent ZW Wilson R Massively parallel digital transcriptional profiling of single cells. Nat Commun (2017) 8:14049. 10.1038/ncomms14049 28091601 PMC5241818

[30] Wang Q Chikina M Zaslavsky E Pincas H Sealfon SC beta-catenin regulates GnRH-induced FSHbeta gene expression. Mol Endocrinol (2013) 27(2):224–37. 10.1210/me.2012-1310 23211523 PMC3683805

[31] Dobin A Davis CA Schlesinger F Drenkow J Zaleski C Jha S STAR: ultrafast universal RNA-seq aligner. Bioinformatics (2013) 29(1):15–21. 10.1093/bioinformatics/bts635 23104886 PMC3530905

[32] Liao Y Smyth GK Shi W. featureCounts: an efficient general purpose program for assigning sequence reads to genomic features. Bioinformatics (2014) 30(7):923–30. 10.1093/bioinformatics/btt656 24227677

[33] Law CW Chen Y Shi W Smyth GK. voom: precision weights unlock linear model analysis tools for RNA-seq read counts. Genome Biol (2014) 15(2):R29. 10.1186/gb-2014-15-2-r29 24485249 PMC4053721

[34] Gentleman RC Carey VJ Bates DM Bolstad B Dettling M Dudoit S Bioconductor: open software development for computational biology and bioinformatics. Genome Biol (2004) 5(10):R80. 10.1186/gb-2004-5-10-r80 15461798 PMC545600

[35] Ritchie ME Phipson B Wu D Hu Y Law CW Shi W Limma powers differential expression analyses for RNA-sequencing and microarray studies. Nucleic Acids Res (2015) 43(7):e47. 10.1093/nar/gkv007 25605792 PMC4402510

[36] Li B Dewey CN. RSEM: accurate transcript quantification from RNA-Seq data with or without a reference genome. BMC Bioinformatics (2011) 12:323. 10.1186/1471-2105-12-323 21816040 PMC3163565

[37] Langmead B Salzberg SL. Fast gapped-read alignment with Bowtie 2. Nat Methods (2012) 9(4):357–9. 10.1038/nmeth.1923 22388286 PMC3322381

[38] Zhang Y Liu T Meyer CA Eeckhoute J Johnson DS Bernstein BE Model-based analysis of ChIP-Seq (MACS). Genome Biol (2008) 9(9):R137. 10.1186/gb-2008-9-9-r137 18798982 PMC2592715

[39] Greene CS Krishnan A Wong AK Ricciotti E Zelaya RA Himmelstein DS Understanding multicellular function and disease with human tissue-specific networks. Nat Genet (2015) 47(6):569–76. 10.1038/ng.3259 25915600 PMC4828725

[40] Heinz S Benner C Spann N Bertolino E Lin YC Laslo P Simple combinations of lineage-determining transcription factors prime cis-regulatory elements required for macrophage and B cell identities. Mol Cell (2010) 38(4):576–89. 10.1016/j.molcel.2010.05.004 20513432 PMC2898526

[41] Yu D Huber W Vitek O. Shrinkage estimation of dispersion in negative binomial models for RNA-seq experiments with small sample size. Bioinformatics (2013) 29(10):1275–82. 10.1093/bioinformatics/btt143 23589650 PMC3654711

[42] van der Maaten L Hinton G Visualizing data using t-SNE. J Mach Learn Res (2008) 9:2579–605.

[43] Amir ED Davis KL Tadmor MD Simonds EF Levine JH Bendall SC viSNE enables visualization of high dimensional single-cell data and reveals phenotypic heterogeneity of leukemia. Nat Biotechnol (2013) 31(6):545–52. 10.1038/nbt.2594 23685480 PMC4076922

[44] Whitfield ML Sherlock G Saldanha AJ Murray JI Ball CA Alexander KE Identification of genes periodically expressed in the human cell cycle and their expression in tumors. Mol Biol Cell (2002) 13(6):1977–2000. 10.1091/mbc.02-02-0030 12058064 PMC117619

[45] Tremblay JJ Drouin J. Egr-1 is a downstream effector of GnRH and synergizes by direct interaction with Ptx1 and SF-1 to enhance luteinizing hormone beta gene transcription. Mol Cell Biol (1999) 19(4):2567–76. 10.1128/MCB.19.4.2567 10082522 PMC84049

[46] Cheng KW Ngan ES Kang SK Chow BK Leung PC. Transcriptional down-regulation of human gonadotropin-releasing hormone (GnRH) receptor gene by GnRH: role of protein kinase C and activating protein 1. Endocrinology (2000) 141(10):3611–22. 10.1210/endo.141.10.7730 11014215

[47] Lawson MA Tsutsumi R Zhang H Talukdar I Butler BK Santos SJ Pulse sensitivity of the luteinizing hormone beta promoter is determined by a negative feedback loop involving early growth response-1 and Ngfi-A binding protein 1 and 2. Mol Endocrinol (2007) 21(5):1175–91. 10.1210/me.2006-0392 17299135 PMC2932486

[48] Lamba P Khivansara V D’Alessio AC Santos MM Bernard DJ. Paired-like homeodomain transcription factors 1 and 2 regulate follicle-stimulating hormone beta-subunit transcription through a conserved cis-element. Endocrinology (2008) 149(6):3095–108. 10.1210/en.2007-0425 18339718 PMC2408822

[49] Suszko MI Antenos M Balkin DM Woodruff TK. Smad3 and Pitx2 cooperate in stimulation of FSHbeta gene transcription. Mol Cell Endocrinol (2008) 281(1–2):27–36. 10.1016/j.mce.2007.10.003 18022758

[50] Purwana IN Kanasaki H Mijiddorj T Oride A Miyazaki K. Induction of dual-specificity phosphatase 1 (DUSP1) by pulsatile gonadotropin-releasing hormone stimulation: role for gonadotropin subunit expression in mouse pituitary LbetaT2 cells. Biol Reprod (2011) 84(5):996–1004. 10.1095/biolreprod.110.088526 21228211

[51] Lamba P Santos MM Philips DP Bernard DJ. Acute regulation of murine follicle-stimulating hormone beta subunit transcription by activin A. J Mol Endocrinol (2006) 36(1):201–20. 10.1677/jme.1.01961 16461939

[52] Xie H Hoffmann HM Meadows JD Mayo SL Trang C Leming SS Homeodomain proteins SIX3 and SIX6 regulate gonadotrope-specific genes during pituitary development. Mol Endocrinol (2015) 29(6):842–55. 10.1210/me.2014-1279 25915183 PMC4447639

[53] Tan NY Khachigian LM Sp1 phosphorylation and its regulation of gene transcription. Mol Cell Biol (2009) 29(10):2483–8. 10.1128/MCB.01828-08 19273606 PMC2682032

[54] Kim JK Kolodziejczyk AA Ilicic T Teichmann SA Marioni JC. Characterizing noise structure in single-cell RNA-seq distinguishes genuine from technical stochastic allelic expression. Nat Commun (2015) 6:8687. 10.1038/ncomms9687 26489834 PMC4627577

[55] Volfson D Marciniak J Blake WJ Ostroff N Tsimring LS Hasty J. Origins of extrinsic variability in eukaryotic gene expression. Nature (2006) 439(7078):861–4. 10.1038/nature04281 16372021

[56] Larson DR Zenklusen D Wu B Chao JA Singer RH. Real-time observation of transcription initiation and elongation on an endogenous yeast gene. Science (2011) 332(6028):475–8. 10.1126/science.1202142 21512033 PMC3152976

[57] Trcek T Larson DR Moldon A Query CC Singer RH. Single-molecule mRNA decay measurements reveal promoter-regulated mRNA stability in yeast. Cell (2011) 147(7):1484–97. 10.1016/j.cell.2011.11.051 22196726 PMC3286490

[58] Zopf CJ Quinn K Zeidman J Maheshri N. Cell-cycle dependence of transcription dominates noise in gene expression. PLoS Comput Biol (2013) 9(7):e1003161. 10.1371/journal.pcbi.1003161 23935476 PMC3723585

[59] Laverriere JN L’Hote D Tabouy L Schang AL Querat B Cohen-Tannoudji J. Epigenetic regulation of alternative promoters and enhancers in progenitor, immature, and mature gonadotrope cell lines. Mol Cell Endocrinol (2016) 434:250–65. 10.1016/j.mce.2016.07.010 27402603

[60] Vallejos CA Risso D Scialdone A Dudoit S Marioni JC. Normalizing single-cell RNA sequencing data: challenges and opportunities. Nat Methods (2017) 14(6):565–71. 10.1038/nmeth.4292 28504683 PMC5549838

[61] Ruf F Park MJ Hayot F Lin G Roysam B Ge Y Mixed analog/digital gonadotrope biosynthetic response to gonadotropin-releasing hormone. J Biol Chem (2006) 281(41):30967–78. 10.1074/jbc.M606486200 16916798

[62] Ruf F Hayot F Park MJ Ge Y Lin G Roysam B Noise propagation and scaling in regulation of gonadotrope biosynthesis. Biophys J (2007) 93(12):4474–80. 10.1529/biophysj.107.115170 17720728 PMC2098712

[63] Sargent LM Dragan YP Sattler G Xu YH Wiley J Pitot HC. Specific chromosomal changes in albumin simian virus 40 T antigen transgenic rat liver neoplasms. Cancer Res (1997) 57(16):3451–6. 9270012

[64] Bloomfield M Duesberg P. Karyotype alteration generates the neoplastic phenotypes of SV40-infected human and rodent cells. Mol Cytogenet (2015) 8:79. 10.1186/s13039-015-0183-y 26500699 PMC4618876

[65] Raser JM O’Shea EK. Noise in gene expression: origins, consequences, and control. Science (2005) 309(5743):2010–3. 10.1126/science.1105891 16179466 PMC1360161

[66] Buenrostro JD Wu B Litzenburger UM Ruff D Gonzales ML Snyder MP Single-cell chromatin accessibility reveals principles of regulatory variation. Nature (2015) 523(7561):486–90. 10.1038/nature14590 26083756 PMC4685948

[67] Cusanovich DA Daza R Adey A Pliner HA Christiansen L Gunderson KL Multiplex single cell profiling of chromatin accessibility by combinatorial cellular indexing. Science (2015) 348(6237):910–4. 10.1126/science.aab1601 25953818 PMC4836442

