# Supplementary material for: Regulatory Architecture of the LβT2 Gonadotrope Cell Underlying the Response to Gonadotropin-Releasing Hormone
Source: Front Endocrinol (Lausanne). 2018 Feb 14;9:34. doi: 10.3389/fendo.2018.00034 (PMC5816955; doi:10.3389/fendo.2018.00034)
Supplement: Supplementary file 1 [file Data_Sheet_1.ZIP › LbT2_RegArchitec_Suppl.Material_rev.docx]

***Supplementary Material***

**Regulatory Architecture of the LβT2 Gonadotrope Cell Underlying the Response to Gonadotropin-Releasing Hormone**

Frederique Ruf-Zamojski, Miguel Fribourg, Yongchao Ge, Venugopalan Nair, Hanna Pincas, Elena Zaslavsky, German Nudelman, Stephanie J. Tuminello, Hideo Watanabe, Judith L. Turgeon, and Stuart C. Sealfon*

* Correspondence: Corresponding Author: [stuart.sealfon@mssm.edu](mailto:stuart.sealfon@mssm.edu)

**SUPPLEMENTARY FIGURES AND TABLES**

**1. Figures**

Suppl. Figs.1 and 2 are provided as separate image files, whereas Suppl. Fig.3 is presented further below.

**Suppl. Figure 1.** High-resolution image version of **Fig. 2A**. Heat map of RNA-seq expression data showing the genes that were differentially regulated following treatment with 5 nM GnRH for 45 min. Gene expression is shown in normalized log2 counts per million (cpm). Differentially expressed genes were selected based on a 4-fold change and FDR < 0.05.

**Suppl. Figure 2.** High-resolution image version of **Fig. 5B**. Heat map of selected genes in all 3881 cells. Each horizontal line is one cell, with red and black on the right side indicating GnRH and vehicle treatment, respectively. Genes symbols are color-coded as follows: red, genes that are differentially regulated by GnRH; black, genes that show high cell-to-cell variability in gene expression; yellow, genes that have been involved in gonadotrope gene regulation based on the literature; blue, housekeeping genes.

**
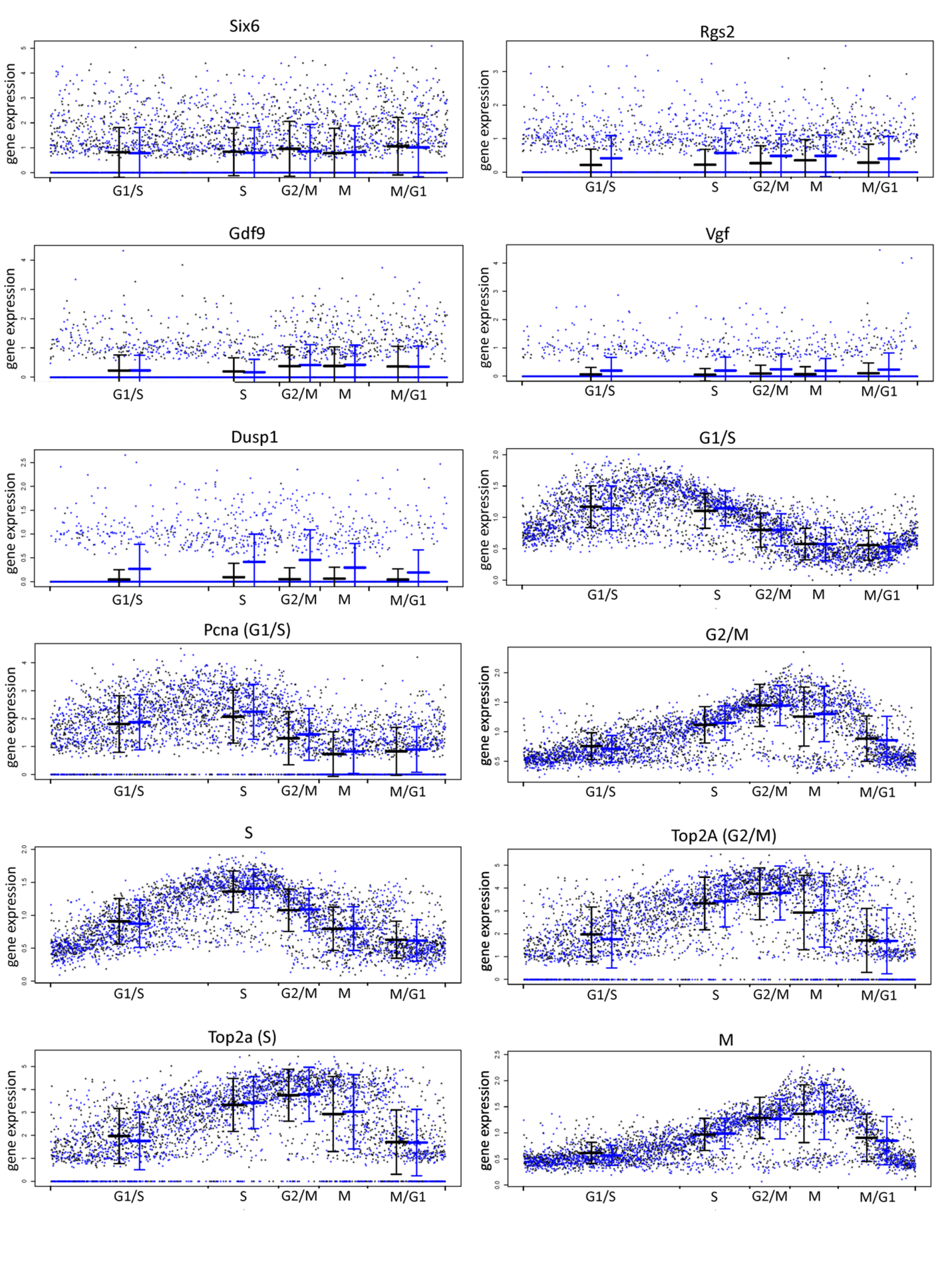
**

**
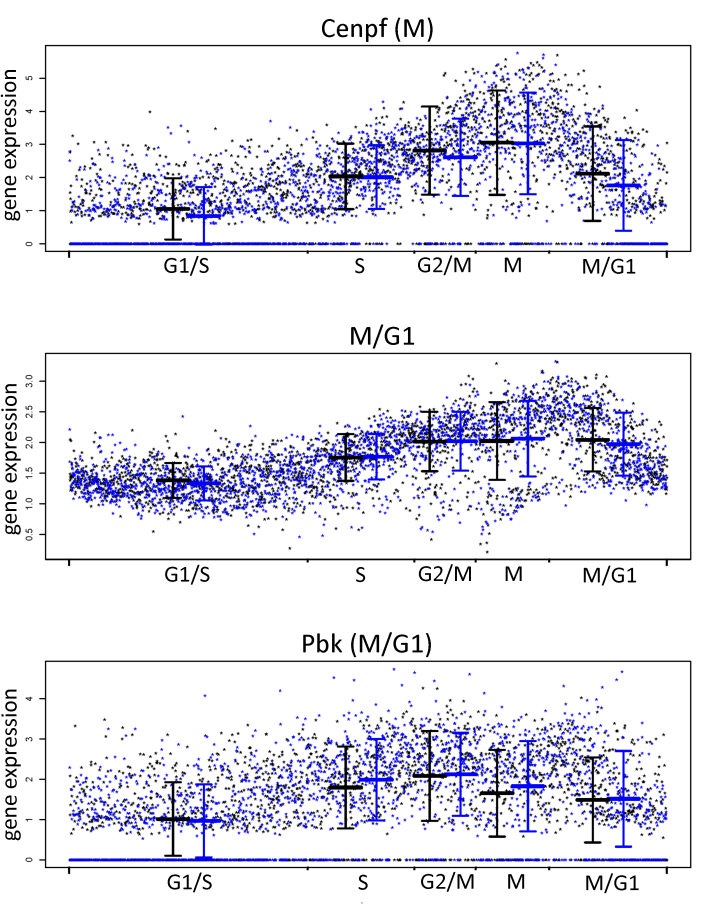
**

**Suppl. Figure 3.** Single-cell expression of individual transcripts in relationship to cell cycle phase. Expression level is plotted as log2 (TPM+1). Several GnRH-regulated genes and genes associated with a specific phase of cell cycle are shown, as indicated. The x-axis indicates cell cycle phase as derived from **Fig. 6B**. The mean and standard deviation for vehicle- (in black) and GnRH-treated cells (in blue) are shown at each cell cycle phase. Note that the expression level of any transcript in these cells at any point in the cell cycle can be accurately determined without experimental cell cycle synchronization and the effect of GnRH on gene induction is shown to be independent of cell cycle phase.

**2. Tables**

Except for Suppl. Table 4 (presented further below), all other tables are provided separately as MS Excel spreadsheets.

**Suppl. Table 1.** List of highly differentially expressed genes in bulk RNA-seq data from GnRH-treated *versus* vehicle-treated LβT2 cells.

**Suppl. Table 2.** List of the candidate genes with the best “edge score values” detected in the GIANT pituitary network

**Suppl. Table 3.** List of GO processes and KEGG pathways that were found enriched in the GIANT pituitary network

**Suppl. Table 4.** List of enriched motifs in genomic regions found in an ATAC-seq sample using the HOMER tool


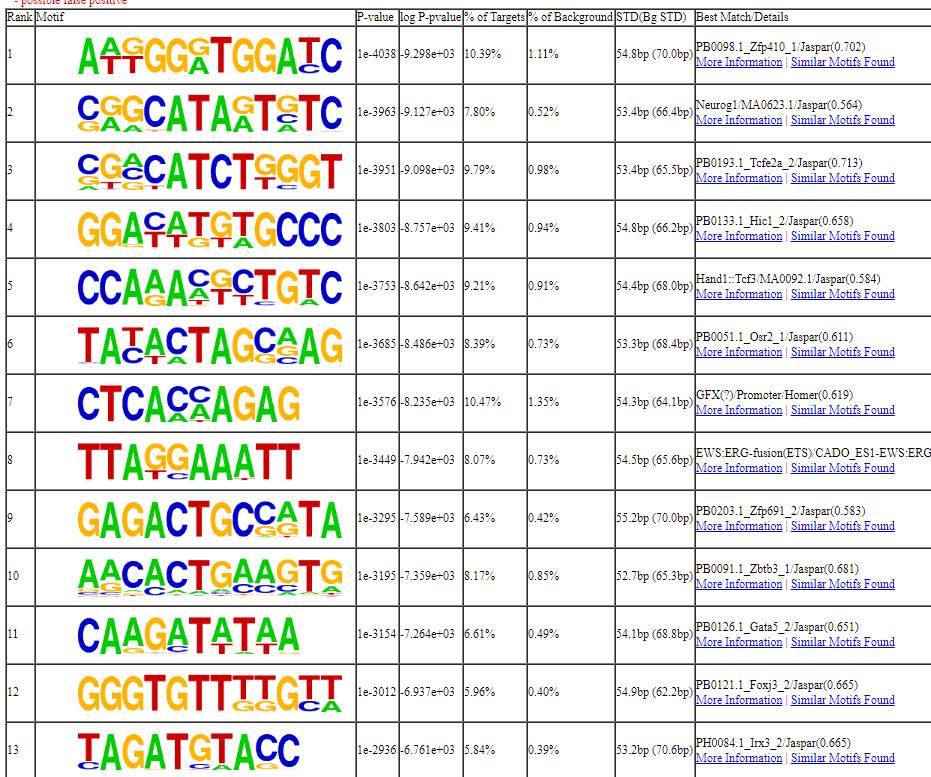


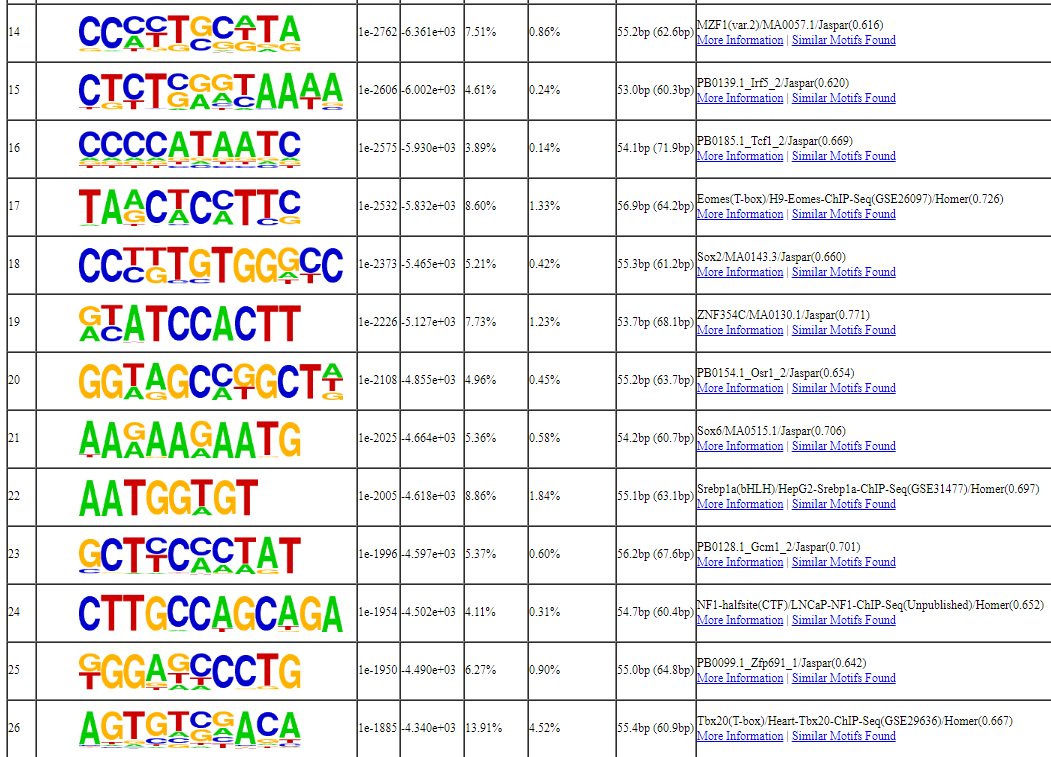


**Suppl. Table 5.** List of differentially expressed genes in GEM Drop-seq data from GnRH-treated *versus* vehicle-treated LβT2 cells

**Suppl. Table 6.** List of cell cycle-regulated genes that were mapped from human to mouse using Homologene
