# Supplementary figures and images for: Regulatory Architecture of the LβT2 Gonadotrope Cell Underlying the Response to Gonadotropin-Releasing Hormone
Source: Front Endocrinol (Lausanne). 2018 Feb 14;9:34. doi: 10.3389/fendo.2018.00034 (PMC5816955; doi:10.3389/fendo.2018.00034)

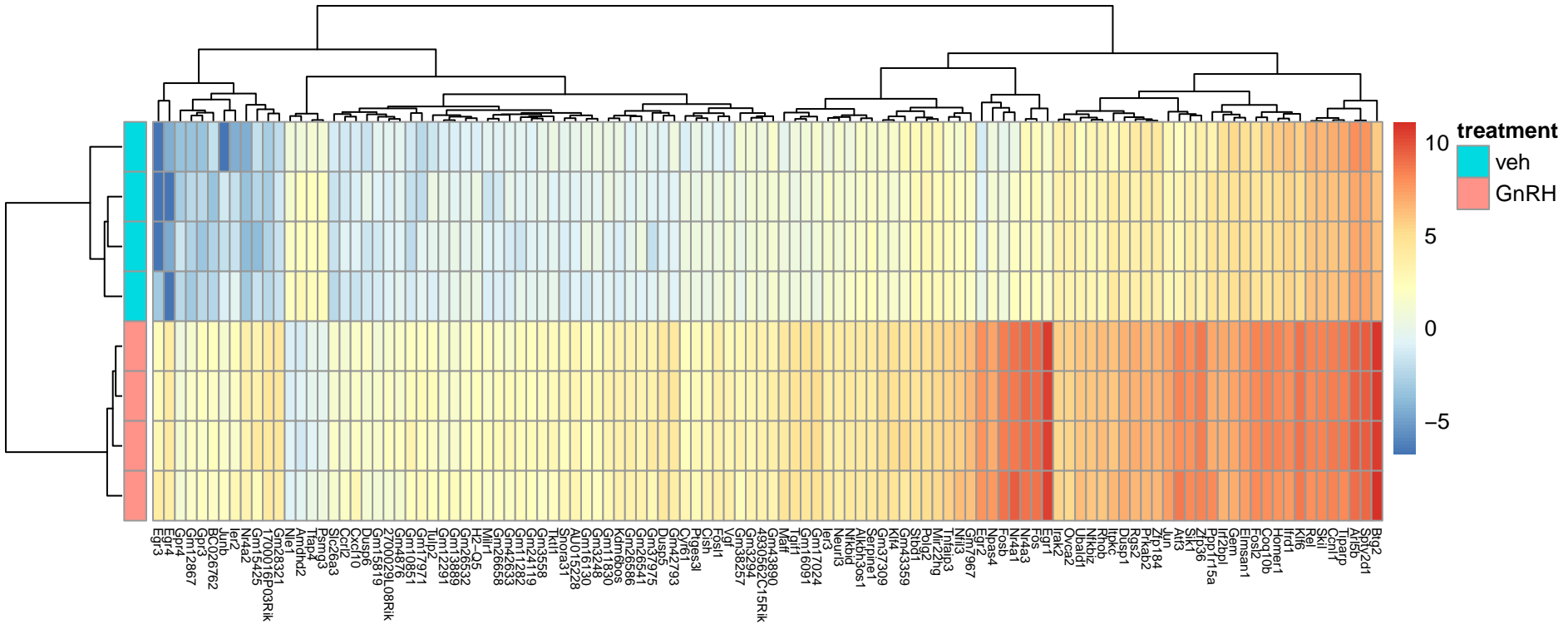

Supplement: Supplementary file 1 [file Data_Sheet_1.ZIP › Suppl.Fig.1.pdf]

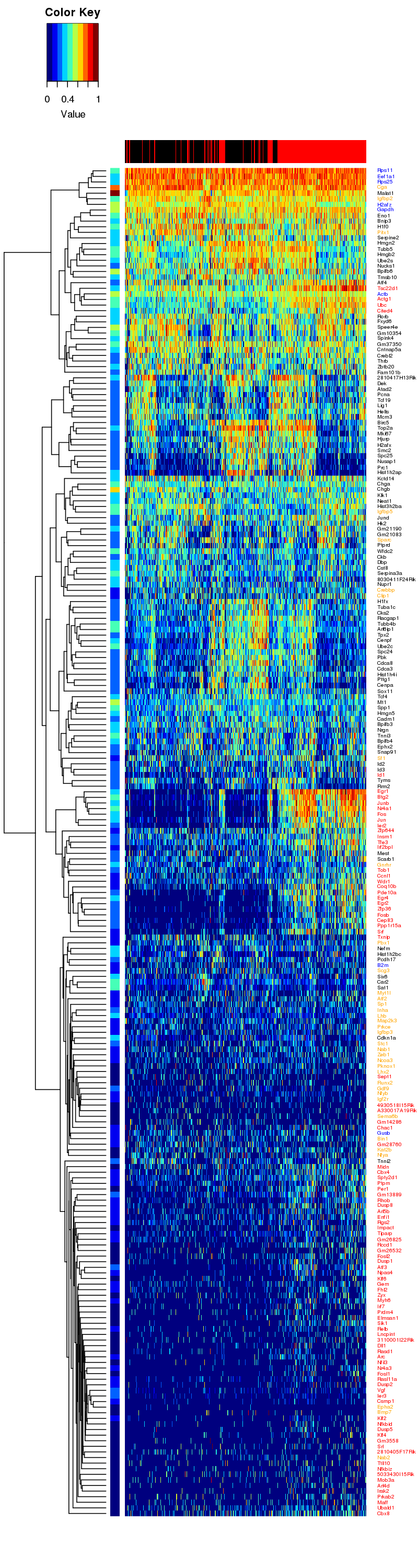

Supplement: Supplementary file 1 [file Data_Sheet_1.ZIP › Suppl.Fig.2.png]
